# Painful considerations in exercise-management for rotator cuff related shoulder pain: a scoping review on pain-related prescription parameters

**DOI:** 10.1186/s12891-025-08411-7

**Published:** 2025-02-22

**Authors:** Kaspar Raulline Ullern, Magnus Richardsen, Ishanka Weerasekara, Bård Erik Bogen

**Affiliations:** 1 Faculty of Health and Social Sciences, Western Norway University of Applied Sciences, Bergen, 5063 Norway; 2 Institute of Health and Wellbeing, Federation University, Churchill, VI 3842 Australia; 3 Department of Global Public Health and Primary Care, University of Bergen, Bergen, 5009 Norway

**Keywords:** Rotator cuff related shoulder pain, Resistance exercise, Scoping review, Pain-related prescription parameters, Pain allowance, Pain intensity, Pain monitoring strategies

## Abstract

**Background:**

Resistance exercise is recommended as the first line of treatment for rotator cuff related shoulder pain (RCRSP), but with conflicting evidence supporting the superiority of specific prescription parameters. Particularly, the role of pain-related prescription parameters remains poorly understood, despite their wide clinical application and potential impact on treatment outcomes. This review aims to investigate how pain-related prescription parameters, such as pain allowance and intensity limits, are reported, described, and applied in clinical trials assessing resistance exercise interventions for RCRSP.

**Methods:**

Guided by PRISMA-ScR, this scoping review followed a comprehensive and systematic search in MEDLINE (Ovid), MEDLINE (EMBASE), Central (Cochrane), PEDro and CINAHL (EBSCO). Two authors independently performed title and abstract screening, and full text screening on eligible records. Randomized clinical trials (RCTs) published in English between 2018 and 2023, applying resistance exercise for RCRSP were included. Both quantitative and qualitative approaches to data analysis were conducted.

**Results:**

The literature search identified 7500 records, of which 4588 titles and abstracts were screened after duplicate removal. Altogether, 304 full texts were screened leaving a total of 86 records in the final analysis. Fifty-eight (67%) studies did not mention the use of any pain-related prescription parameters, resulting in data extraction from the 28 remaining studies. Applied parameters were widely heterogenic, but three categories of pain allowance styles were identified and categorized into “yes”, “no” or “ambiguous”. These categories were commonly guided by specific Numerical Rating Scale (NRS)/Visual Analog Scale (VAS) limits or individual pain tolerance, used for pain monitoring and exercise progression. Citations and/or justifications for the chosen pain-related prescription parameters were reported by 10 (36%) studies, in which 5 main themes for justifications, and 3 key papers for the citations were identified.

**Conclusion:**

This review reveals substantial reporting deficiencies regarding pain-related prescription parameters in RCTs addressing RCRSP with resistance exercise. The identified parameters varied widely, reflecting a lack of consensus and evidence-based guidance in the literature and in a clinical setting. To advance our understanding on the role of pain-related prescription parameters, more consistent reporting of these parameters in future research is warranted.

**Trial registration:**

Published on the Open Science Framework 28.02.24: osf.io/a52kn.

**Supplementary Information:**

The online version contains supplementary material available at 10.1186/s12891-025-08411-7.

## Introduction

Shoulder pain is commonly reported as the third most prevalent musculoskeletal pain presentation in primary care [1], with a considerable financial burden due to associated sick leave and healthcare costs [2]. In the general, community based population, prevalence rates of shoulder pain are reported with a median of 16% (range 0.67–55.2%), and with a tendency to increase with increasing age [3]. It is presumed that rotator cuff related shoulder pain (RCRSP) accounts for approximately 70–80% of consultations involving shoulder pain [4, 5].

RCRSP is used as a diagnostic term for clinical presentations of weakness and pain in the shoulder, typically during shoulder movements such as external rotation and shoulder elevation [6]. Recent clinical practice guidelines describe RCRSP as an umbrella term encompassing a spectrum of various shoulder conditions including rotator cuff tendinopathy, subacromial pain syndrome, calcific tendinopathies, and partial and/or age-related tears in the rotator cuff [7]. Despite the large amount of “special” orthopedic shoulder tests available, there is no definitive way to isolate individual structures in the shoulder while ruling out that pain may come from nearby structures [8]. In line with the high prevalence of radiological shoulder pathologies in asymptomatic populations [9, 10], and a lack of correlation between pain and structural factors [11], the omission of a definitive structural pathognomonic label may be appropriate [6]. Consequently, based on a pragmatic approach, the term RCRSP has been used in this review.

Multiple treatment approaches for RCRSP exist, such as surgeries, medications, electrotherapy, injections, manual therapies, exercises, and education [12]. However, a shift from invasive and passive treatment modalities to exercise therapy as the primary treatment option for RCRSP has been advocated in recent years [13, 14]. Both current clinical practice guidelines and surveys of current physiotherapy practice for the treatment of RCRSP reflect this paradigm shift [7, 15]. Among exercise interventions, progressive resistance exercise has been shown to be a well-documented intervention for the improvement in pain and function outcomes for RCRSP [16]. However, there is a lack of evidence supporting the superiority of specific exercise prescription parameters [4, 14, 16, 17].

Expert consensus has revealed different attitudes about how pain during exercise for RCRSP should be managed and interpreted [18]. From a patient perspective, experiencing shoulder pain during exercise has been shown to act as a barrier, but also as a facilitator for exercise adherence, reflecting the complexity of achieving standardized optimal load management [19, 20]. Furthermore, existing evidence shows associations between an optimized therapeutic alliance, self-efficacy, and contextualization of pain during exercise as predictors for favorable treatment outcomes and behaviour change [21, 22]. A systematic review investigating treatment outcomes for chronic musculoskeletal pain disorders, concludes that in the short term, pain allowance during exercise may provide a slight beneficial effect on pain and function, compared to disallowing painful exercise [23]. Similarly, another review article concludes that exercises with and without pain are both effective for function, shoulder pain, and range of motion, without significant differences [17]. However, these conclusions are based on small study samples.

Insufficient reporting of exercise interventions and participant characteristics in randomized controlled trials (RCTs) on RCRSP has led to uncertainty about effective exercise interventions and their components, limiting clinical transferability [24]. The Consensus on Exercise Reporting Template (CERT) was developed in 2016 to improve research transparency, facilitating replication and implementation of effective exercise interventions into clinical practice [25]. Despite these efforts, recent systematic reviews call for more details on all components of exercise interventions and consistent use of reporting guidelines [26, 27].

Pain-related prescription parameters, like pain allowance, applied pain limits and progression guided by pain in exercise prescription have been given little attention in previous research on RCRSP [28, 29]. This dearth of evidence stands in contrast to the frequent use of these parameters to inform exercise prescription in a clinical context [30, 31]. Given the scientific and clinical uncertainty regarding pain-related prescription parameters and their role in RCRSP rehabilitation, this scoping review aims to explore the applications of these parameters in exercise interventions for RCRSP by mapping the latest research within the field. To facilitate and clarify the purpose of this review, the following research question and subsequent objectives were developed, using the *population*,* context and concept* (PCC) framework [32], which is illustrated in Table 1.

Primary research question:

How are pain-related prescription parameters reported, described, and applied in clinical trials using resistance exercise interventions for RCRSP?

Research objectives:

1. To explore how pain-related prescription parameters like pain allowance and pain monitoring models are used to guide symptom management and exercise dosage in RCRSP.
2. To explore the underlying justifications and supporting evidence used to guide the choice of pain-related prescription parameters in the included trials.

## Methods

### Theoretical framework

This scoping review is grounded in the international methodological recommendations by Levac et al. [33] and Peters et al. [32], to maximize the relevance of our findings, ensure methodological quality and minimize research redundancy. We adhered to the PRISMA-ScR (Preferred Reporting Items for Systematic reviews and Meta-Analyses extension for Scoping Reviews) checklist to ensure methodological transparency and completeness of reporting [34].

### Study protocol and registration process

Joanna Briggs Institute’s (JBI) Manual for Evidence Synthesis [35], and the PRISMA-ScR Checklist [34] was used to prepare the protocol. This scoping review’s protocol was published on the Open Science Framework (OSF) on February 28, 2024, and can be accessed at *osf.io/a52kn*.

### Search strategy

A limited search in MEDLINE and CINAHL was performed to establish an overview of existing research, leading to the identification of relevant knowledge gaps. Text words from titles, abstracts, and index terms were collected and later informed the development of the primary research question and database search, in accordance with recommendations from the “JBI Manual for Evidence Synthesis” [35]. Additionally, we performed a search in the databases OSF and Prospero to explore preregistrations on similar topics to minimize the risk of research redundancy.

We have chosen to follow JBI’s guidance by using the PCC-framework to identify and define the main concepts in our primary review question [32]. Based on the terms and concepts provided from the preliminary search, we developed the foundation of our research question, illustrated in Table 1.

**Table 1:** Primary research question in a PCC-format

PCC component	Research question components
(P)opulation	Individuals aged 18 + with clinically diagnosed RCRSP.
(C)oncept	Pain-related prescription parameters in resistance exercise interventions. This includes instructions on pain allowance, pain intensity measured with or without pain scales, pain monitoring models, dosage and progression/regression of exercise load determined by pain response and reported prescription/use of analgesics.
(C)ontext	RCTs (pilot and feasibility studies included) involving one or several exercise interventions for RCRSP, published in the last five years (1 jan 2018–5 dec 2023).

Eligibility criteria (Table 2) were developed during the preparation phase and preliminary search, and subsequently adapted in concordance with the PCC-framework [36].

**Table 2:** Eligibility criteria

Criteria for inclusion	• Age: 18 + • Clinically diagnosed RCRSP or any diagnostic label coinciding with RCRSP. E.g.: rotator cuff tendinopathy, subacromial pain syndrome, subacromial impingement syndrome, subacromial bursopathy, long head biceps tendinopathy, and partial thickness rotator cuff tear [7, 37] • Study design: RCTs, or pilot/feasibility studies with a RCT design. • Resistance exercise intervention exclusively or in conjunction with any other treatment(s) (a pragmatic approach to defining “resistance exercise” was employed, based on definitions by ACSM’s Resource Manual for Guidelines for Exercise Testing and Prescription [38], and Pavlova et al. [27]).
Criteria for exclusion	• Other languages than English • Patients with cancer, fibromyalgia, inflammatory disease, history of shoulder disclocation, subluxation or chronic instability, cardiovascular surgery, rheumatoid arthritis, prosthesis, arthroplasty, previous shoulder surgery, corticosteroid injection, full-thickness tear, massive rotator cuff tear, frozen shoulder, severe osteoarthritis of the glenohumeral joint, fracture, neurological disorders. • Exercise modalities not meeting our defined criteria for resistance exercise (E.g. only stretching and/or mobility exercise)

Experimental studies with an RCT-design, written in English and published between 2018 and the date of search (1 jan 2018–5 dec 2023) were deemed eligible. As the gold standard in clinical experimental research [39], RCTs are highly influential in evidence-based decision-making. Consequently, a limitation to experimental studies (including pilots and feasibility studies) with an RCT design was deemed appropriate, considering the aim of this review. Given the increased focus on reporting and transparent research conduct in recent years [25], we wanted to examine the latest research in the field. Therefore, a decision was made to only extract data from studies published from 2018 onwards. A filter starting from 2013 was applied in the database search, to include study registries submitted before 2018, for the scenario where studies were indexed with multiple date entries [40].

Because RCRSP is a broad definition and is referred to as an overarching diagnostic label - encompassing multiple anatomical structures - pragmatic considerations were deployed in the development of diagnosis-specific eligibility criteria. Hence, a variety of diagnostic labels were accepted for inclusion, but clear exclusion criteria were also applied based on the latest nomenclative delimitation [7, 37]. We considered any exercise with an external load greater than or equal to active movement against the force of gravity as resistance exercise. Studies only containing interventions described as stretching or mobility exercise (with the force of gravity eliminated), were consequently excluded. To elaborate on the exclusion criteria regarding corticosteroid injections: studies were included if injections were not administered to all intervention arms, as the prescription parameters for exercise in the injection-free arms could provide valuable insights. Conversely, studies where all intervention arms with an exercise component had received injections were excluded, as the prescription parameters might have been influenced by the effects of the injection prior to exercise.

A strategic selection of electronic databases was conducted to meet methodological requirements and ensure comprehensive coverage of research within musculoskeletal disorders [41]. The following databases were utilized in the search: MEDLINE (Ovid), MEDLINE (EMBASE), Cochrane (CENTRAL), PEDro, and CINAHL (EBSCO). The final search was developed in MEDLINE (Ovid) and was then adapted to the remaining databases. A summary of the search strategy for all included databases can be found in “Additional file 1”. We chose to include a broad range of search terms to ensure sufficient research coverage of the field, as is customary within the framework of scoping reviews [42]. Different databases were cross searched for key papers to optimize search strategy validity across our chosen databases. Before conducting the final search, a librarian at Western Norway University of Applied Sciences with experience in this methodological approach, conducted a review of the literature search and translations to other databases. The final and concluding update of the search was carried out on December 5, 2023. Only RCTs were included in this review, and no supplementary search for grey literature was performed due to the large number of papers yielded from the database search.

### Study selection/screening process

Covidence systematic review manager software was used as a tool for duplicate removal, selection and extraction of the included studies following the final search. Covidence allowed for independent screening ensuring blinding throughout the entire screening process, as well as organized resolving of conflicts with the option to add external collaborators.

Throughout the screening process, the *JBI Manual for Evidence Synthesis* [35] was followed as a guide to minimize risk of bias. The screening process consisted of 3 stages: (1) pilot screening, (2) Title & abstract screening, and (3) Full text screening. Each screener evaluated a randomly selected sample of 25 titles/abstracts and then screeners convened to discuss possible modifications or discrepancies. When consensus of 75% or more was reached and a common assessment basis for inclusion and exclusion criteria was established, the screening process continued separately with the screeners blinded to each other [32]. This step was conducted for both the title and abstract screening, and full text screening to optimize reliability throughout the process. Disagreements in the screening process were sorted under “conflicts” in Covidence and resolved through discussion. In cases of uncertainty beyond discussion a third collaborator was invited to resolve conflicts.

For all studies remaining after the screening process, an additional step was applied to maximize the relevancy of studies included for data extraction. Studies that did not describe or report any form of pain-related prescription parameters, were excluded from further data extraction as they would provide minimal relevant data to answer our research question. A reference list of these excluded studies is provided in “additional file 2”. We considered any information in the full-text articles, including supplementary material and appendixes that could be related to, or imply the application of any pain-related prescription parameters as appropriate for inclusion.

### Data charting

An iterative approach for the data charting based on recommended guidelines [36], was conducted in several stages: (1) A draft extraction form based on the PCC-framework was developed in an Excel spreadsheet. (2) Pilot testing of the draft extraction form was done separately by both reviewers on 3 different articles. (3) Following the pilot testing, a group discussion with both reviewers and a counsellor with expertise on data extraction in scoping reviews took place. Agreement was reached regarding the different aspects of the extraction tool. Consequent to discussion, a refined data extraction template was created in Covidence, along with extraction guidance for each data item. The data item categories from the final extraction template are summarized in Table 3 below. (4) Only the data categories covering pain-related prescription parameters were extracted separately by both reviewers to minimize extraction bias for data with the highest relevancy. For all data concerning study characteristics and intervention descriptions, we deemed that one extractor would be sufficient as those data categories were extracted on a less interpretive basis. (5) Finally, extractors convened to resolve disagreements and dissimilarities in the extracted data. If no agreement could be reached, a third collaborator was invited to resolve the conflict. When consensus was reached for all the data categories in all studies, the final data material was exported from Covidence into an excel spreadsheet for data analysis.

The iterative approach allowed for continuous refinement of the extraction template in the earlier stages, which helped develop additional categories for data charting throughout the data charting process.

**Table 3:** Summary of data items*

Study characteristics	• Author(s), title, country of origin, publication year • Study design, purpose, study setting • Diagnostic label(s), inclusion/exclusion criteria • Participant demographics
Intervention descriptions	• Types of interventions • Intervention parameters (duration, setting, frequency, intensity, progression criteria) • Intervention group demographics
Pain-related prescription parameters	• Pain allowance prescription styles • Description of given pain-related instructions • Pain monitoring methods, limits and values • Rationale and citations underpinning the chosen instructions • Analgesic use and group distribution

*Data item categories were adapted based on the main research question, within a PCC-framework

### Data analysis

The extracted data was grouped into logical categories with properties to answer the research question. The process involved a collective, descriptive analysis of extracted data, looking for contrasts or commonalities to identify recurring patterns. Counts of frequency were performed on categories like numerical data, binary data and predefined themes that allowed standardization of certain values.

Basic qualitative content analysis was conducted on textual data related to justifications and rationale provided for the different pain-related prescription parameters. An inductive analytical approach was undertaken in line with Pollock et al.’s [36] recommendations for qualitative analysis in scoping reviews, and the data was synthesized narratively. This process was done collectively by both authors of this review by listing initial thoughts, identifying potential categories through open coding and ultimately establishing the final categories. To identify influential papers on pain-related prescription parameters, backwards citation tracking was performed on all the included studies providing citations for their chosen pain-related prescription parameters [43, 44].

In line with methodological guidelines for scoping reviews, systematic critical appraisals of study results or assessments of the trustworthiness of data documentation in included studies were not conducted [35], however each study was rated according to their respective PEDro scores for the reader’s interest [see Additional file 3].

## Results

**Fig. 1:**
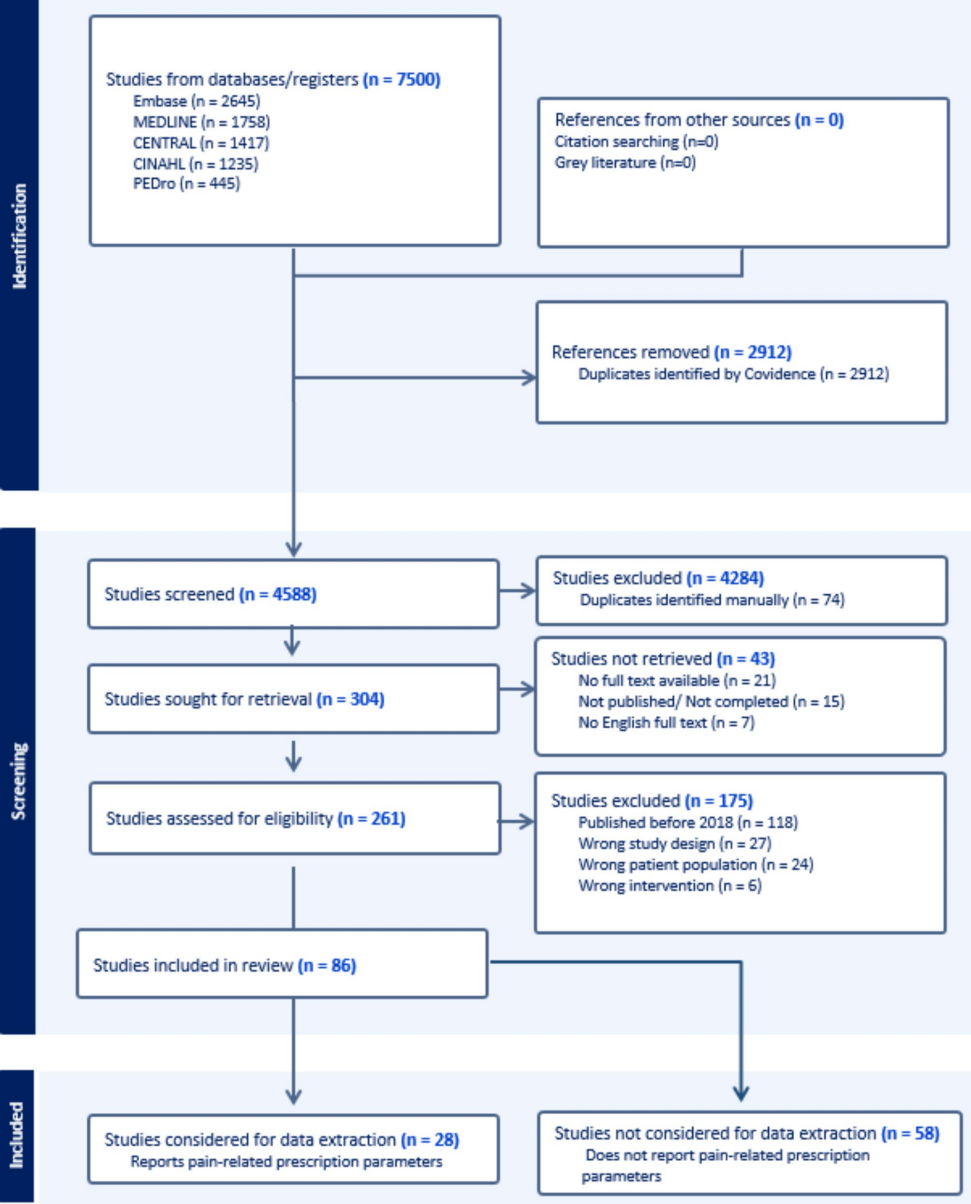
Preferred Reporting Items for Systematic Reviews and Meta-Analyses (PRISMA) flow chart The screening process with reasons for removal and exclusion of studies is presented in Fig. 1 as a PRISMA flow chart

The database search yielded 7500 potential studies, and after automatic duplicate removal, 4588 relevant studies remained for title and abstract assessment. Altogether, 304 studies passed the predefined eligibility criteria based on title and abstract screening, and 261 English full texts were retrieved. Reasons for removal and exclusion of studies are presented in Fig. 1 as a PRISMA flow chart. A total of 86 studies passed the predefined eligibility criteria during full text screening. Interestingly, 58 (67%) of these studies did not report the use of any pain-related prescription parameters. Since these studies would provide minimal relevant data to answer our research question, full data extraction was only carried out on the rest of the studies in which any pain-related prescription parameters were reported. A total of 28 (33%) studies, all published after 2018 were therefore included for data analysis. An overview of study characteristics for these 28 studies is presented in Table 4.

**Table 4:** Study characteristics

Lead author (year), country	Study design	Diagnosis	Total sample size (analyzed)	Mean age of all groups	Symptom duration	Intervention type (group number)	Intervention duration	Use of analgesics
Ager et al. [45] (2019), Canada	Pilot RCT	Rotator cuff tendinopathy	31*	35.1	(1) §§: 23.2 ± 41.5 (2) §§: 38.9 ± 50.5	(1) Neuromuscular training program (2) Usual Physiotherapy Care	6 weeks	N/R
Belley et al. [46] (2018), Canada	RCT	Rotator cuff tendinopathy	40*	45.5	(1) §: 41 ± 35 (2) §: 48 ± 31	(1) Real Anodal Transcranial Direct-Current Stimulation (a-tDCS) + sensorimotor training, strengthening, patient education, home exercises (2) Sham a-tDCS Group + same program as group 1	12 weeks	N/R
Berg et al. [47] (2021), Norway	RCT	Subacromial Pain Syndrome	21**	48.5	> 3 months	(1) high-intensity aerobic training (HIIT) of RC + usual care (2) standardised usual care	8 weeks	N/R
Boudreau et al. [48] (2019), Canada	RCT	Rotator cuff tendinopathy	39**	49.9	> 4 weeks	(1) RC strengthening (2) RC strengthening + pectoralis and latissimus coactivation	6 weeks	Allowed
Clausen et al. [49] (2021), Denmark	RCT	Subacromial impingement	200**	50.5	> 3 months	(1) Usual care + “Strengthen Your Shoulder’’ + home-based progressive high-volume resistance training. (2) Usual care	4 months	Mild pain medication allowed, strong pain medication (excluded)
deOliveira AKA et al. [50] (2022), Brazil	RCT	Subacromial pain syndrome	24*	N/R	> 8 weeks	(1) Biofeedback Group + Strengthening exercise (2) Strengthening exercise	8 weeks	N/R
deOliveira FCL et al. [51] (2021), Canada	RCT	Rotator cuff related shoulder pain	52*	30.2	(1) §§: 20.6 ± 27.7 (2) §§: 24.6 ± 25.7	(1) Kinesio tape + exercise (2) Exercise	6 weeks	Allowed
Dubé et al. [52] (2023), Canada	RCT	Rotator cuff related shoulder pain	123 *	47.7	> 3 months	(1) Education (2) Strengthening exercise (3) Motor control training	12 weeks	N/R
Eliason et al. [53] (2021), Sweden	RCT	Subacromial pain syndrome	120*	44.9	> 4 weeks − 1 year	(1) Joint mobilization + guided exercises (2) Guided exercises (3) CG (no treatment)	12 weeks	Allowed
Eraslan et al. [54] (2023), Turkey	RCT	Subacromial Pain Syndrome	34*	29	> 6 weeks	(1) Scapula exercise + manual therapy (2) Scapula retraction + glenohumeral rotational exercise + manual therapy (3) CG	12 weeks + 12 additional weeks (home program)	NSAIDs not allowed
Gomes et al. [55] (2018), Brazil	RCT	Shoulder impingement syndrome	45*	48.0	> 3 months	(1) Exercise + Manual Therapy (2) Exercise + Manual Therapy + IFC. (3) Exercise + Manual Therapy + Placebo Ultrasound	8 weeks	N/R
Gutiérrez-Espinoza et al. [56] (2019), Chile	RCT	Subacromial pain syndrome:	80*	44.9	> 3 months	(1) Exercise program (2) Exercise program + stretching	12 weeks	Prescribed as part of intervention
Gutiérrez-Espinoza et al. [57] (2023), Chile	RCT	Subacromial impingement syndrome	72*	44.9	> 3 months	(1) Exercise program + Scapular mobilization (2) Exercise program	6 weeks	Prescribed as part of intervention
Hopewell et al. [58] (2021), England (UK)	RCT	Rotator cuff disorders	708*	55.5	< 6 months	(1) Best practice advice (2) Corticosteroid injection + Best practice advice (3) Progressive exercise (4) Progressive exercise + Corticosteroid injection	12 months	Allowed
Hui et al. [59] (2023), China	Pilot RCT	Subacromial pain syndrome	54*	47.4	< 3 months	(1) Yi Jin Bang exercise (2) Exercise therapy (3) CG	10 weeks	N/R
Juul-Kristensen et al. [60] (2019), Denmark	RCT	Subacromial pain syndrome	49 *	43.0	< 4 months	(1) Neuromuscular exercise + EMG-feedback (2) Neuromuscular exercise	8 weeks	N/R
Kang et al. [61] (2019), Taiwan	RCT	Impingement syndrome	36 *	47.5	N/R	(1) Kinesio taping + Exercise (2) Placebo kinesio taping + Exercise	4 weeks	N/R
Karaaslan et al. [62] (2023), Turkey	RCT	Subacromial impingement syndrome	50 *	45.9	(1) §§: 6.75 ± 1.87 (2) §§: 6.95 ± 1.94	(1) Exercise + NMES, (2) Exercise	8 weeks	N/R
Kim et al. [63] (2020), Korea	RCT	Subacromial impingement syndrome	26*	46.1	> 3 weeks	(1) Neurac technique (2) Manual therapy	4 weeks	N/R
Letafatkar et al. [64] (2021), Iran	RCT	Shoulder impingement syndrome	120 *	37.8	> 6 weeks	(1) Therapeutic exercise (2) Therapeutic exercise + Kinesio tape (3) CG	8 weeks	Allowed
Macías-Hernández et al. [65] (2021), Mexico	Pilot RCT	Rotator cuff injury (partial tear)	26 *	54.3	> 3 months	(1) Eccentric exercise (2) Concentric exercise	12 months	N/R
Malliaras et al. [66] (2020), Australia	Feasibility RCT	Rotator cuff related shoulder pain	36	53.8	> 3 months	(1) Advice only (2) Recommended care (3) Recommended care + strengthening exercise	12 weeks	Allowed
Martins da Silva et al. [67] (2020), Brazil	RCT	Rotator cuff tendinopathy	57**	48.3	> 3 months	(1) Kinesio tape (2) Exercise + kinesio tape (3) Exercise only	4 weeks	N/R
Ribeiro et al. [68] (2022), New Zealand	Feasibility RCT	Subacromial pain syndrome	28*	43.9	Total mean §§: 49 ± 76.3	(1) Tailored exercise + MT group (2) Standardized exercise programme	8 weeks	Allowed
Rodriguez-Huguet et al. [69] (2020), Spain	RCT	Supraspinatus tendinopathy	36**	40.1	N/R	(1) Percutaneous Electrolysis + Eccentric exercise (2) Dry needling + Eccentric exercise	4 weeks	Allowed
Santello et al. [70] (2020), Brazil	RCT	Rotator cuff tendinopathy	60*	53.5	> 3 months	(1) Home-based exercise (2) Advice on symptom management	8 weeks	Allowed
Subbiah et al. [71] (2023), India	RCT	Subacromial impingement syndrome	97	45.0	< 3 months	(1) Manual therapy + Eccentric + Specific exercise (2) Conventional exercise	12 weeks	N/R
Vallés-Carrascosa et al. [72] (2018), Spain	RCT	Subacromial pain syndrome	22*	58.5	(1) §§: 12 ± 3.06 (2) §§: 8.0 ± 2.04	(1) Strengthening + stretching exercise (Pain allowed) (2) Strengthening + stretching exercise (No pain allowed)	4 weeks	N/R

Abbreviations: RCT: Randomised controlled trial, N/R: Not reported, CG: Control group, RC: Rotator cuff, *: Intention-To-Treat (ITT) analysis, **: Per-Protocol-Analysis (PPA), §: symptom duration in weeks (including the ± SD), §§: symptom duration in months (including the ± SD)

### Presentation of included studies

Altogether 28 RCTs, originating from 17 different countries were included. The total sample size was 2326 participants, with a total mean age of 45.9 years. A variety of diagnostic labels were identified, with subacromial pain syndrome being the most common, reported by nine studies [47, 50, 53, 54, 56, 59, 60, 68, 72]. Ten studies [47, 49, 52, 55–57, 65–67, 70] only recruited participants with symptoms lasting over three months, while eight studies [45, 46, 51, 61, 62, 68, 69, 72] did not specify any requirement for duration of symptoms to be included in their studies. Symptom duration requirements for the remaining studies [48, 50, 53, 54, 58–60, 63, 64, 71] were heterogenic, and can be found in Table 4. There were also large variations in the duration of exercise interventions, ranging from 4 weeks to 1 year. There was considerable heterogeneity in the included exercise interventions with regards to imposed loading intensities, but all studies included a resistance exercise component meeting the predefined inclusion criteria. Only 13 (46%) studies [48, 49, 51, 53, 54, 56–58, 64, 66, 68–70] reported information regarding usage or prescription of analgesics.

### Pain allowance prescription styles

Three different approaches to pain allowance during exercise were identified and categorized into: “yes”, “no” and “ambiguous”. Altogether, 12 studies (43%) [45, 47–49, 52, 53, 58, 59, 61, 66, 69, 71] specified that some degree of pain was allowed during exercise and were therefore categorized as “yes”. Six studies (21%) [46, 54–57, 70] were categorized as “no”, as they explicitly discouraged exercising with pain. For the 10 remaining studies (36%) [50, 51, 60, 62–65, 67, 68, 72], no firm conclusion could be made for the classification into “yes” or “no”, leading to their chosen approach being categorized as “ambiguous”. An example of a study categorized as “ambiguous” is Karaaslan et al. [62], providing the following instructions in their study: *“All patients in both groups were taught to perform the exercises at maximum pain-free ROM”* and *“The exercises were planned according to the patient’s tolerance”.* Another study classified as “ambiguous”, by Vallés-Carrascosa et al. [72], applied different approaches to different intervention groups (“yes” and “no”). Among the 28 studies analysed in this review, this was the only study designed to compare the effectiveness of two different pain-related prescription parameters.

### Pain monitoring instruments

Numerical Rating Scale (NRS), applied in six (21%) of the included studies [45, 48, 50, 66, 68, 69] was the most used instrument for pain monitoring during exercise, followed by Visual Analogue Scale (VAS), applied in four (14%) studies [53, 61, 71, 72]. Two (7%) studies [47, 52] reported numerical pain limits without further specification regarding which scale they used. Pain levels of 3/10 (applied in 14% of the studies) [45, 52, 66, 69] and 4/10 (applied in 14% of the studies) [47, 48, 53, 72] were the most commonly used numerical pain limits, followed by 5/10 (applied in 7% of the studies) [61, 71] regardless of pain scale. Ribiero et al. [68] specified that an increase of pain by three points in relation to habitual pain was permissible during exercise, and was the only study using a dynamic upper numerical limit. Individual pain tolerance was utilized as the sole pain allowance regulation in five (18%) of the included studies [58, 60, 62, 63, 65], while three (11%) studies [52, 66, 68] applied a mix of individual pain tolerance and numerical pain limits. Individual pain tolerance comprised instructions such as “up to individual pain tolerance”, and “individually manageable pain”.

### Timing of given pain-related instructions

The timing of pain-related instructions was most commonly applicable while the exercises were performed, an approach used in 15 (54%) studies [45, 46, 48, 51, 54–57, 60, 62–64, 69, 70, 72]. This timing-approach was evenly applied for all pain allowance prescription styles (“yes”, “no” and “ambiguous”). For seven (25%) studies [58, 59, 61, 65, 66, 68, 71], the pain-related instructions were applicable both during and after exercise. Two studies (7%) [49, 52] exclusively applied their pain-related instructions based on the symptomatic response after an exercise-session, regardless of pain-intensity levels during exercise. This included instructions like “no increased pain lasting more than 24 hours” [49] and ”any pain is permissible as long as there is no exacerbation of pain in the evening and the following day” [52]. Pain-related instructions based on pain intensity both before and during exercise were applied in two (7%) studies [47, 50]. Furthermore, two (7%) studies [53, 67] applied their pain-related instructions both before, during, and after exercise.

### Pain as a parameter used to inform exercise-progression and dosage

Of the studies included in this review 22 (79%) [45–56, 60, 62, 63, 66–71] used pain intensity or pain response as a parameter to guide load- and exercise progression. Pain-related prescription parameters applied before, during, and after exercise was utilized as an active clinical tool for continuous load adjustment, but with varying approaches. The most commonly used pain-related progression criterion was increased load or progression to next phase when the exercise could be performed free of pain, applied by nine (32%) of the included studies [46, 48, 51, 54–56, 63, 67, 71]. This progression criterion was utilized by studies allowing some degree of pain during exercise, as well as by studies that discouraged pain during exercise. For five (18%) studies [47, 60, 62, 65, 66], it was specified that load-progression could occur despite some pain during exercise, based on individual tolerance or specific pain limits. In the event of pain intensity during exercise exceeding the prescribed pain limits, reduction of the exercise-load was carried out in five (18%) studies [45, 50, 66, 68, 69]. A similar approach was employed in four (14%) studies [49, 52, 53, 68], where load was reduced in the next session if participants experienced sustained exacerbation of symptoms during or after exercise. Correspondingly, Dubé et al. [52] considered the absence of sustained exacerbation of symptoms after exercise as a cue to increase the load in the subsequent exercise session. Two studies (7%) [68, 70] provided instructions to terminate or switch exercises in the event of exceeding prescribed pain limits. In total, four (14%) studies [48, 52, 66, 68] applied a mixed approach, consisting of two or more of the aforementioned pain-related conditions informing regression or progression of load.

### References and justifications underpinning the pain-related prescription parameters

Ten (36%) studies provided some form of justification [65], citation [53, 56, 68] or a combination of both [47, 49, 52, 58, 66, 72] for the use of their respective pain-related prescription parameters. We considered any justification or citation(s) reported in the full-text articles, including supplementary material and appendixes, that was explicitly related to the chosen pain-related prescription parameter.

**Fig. 2:**
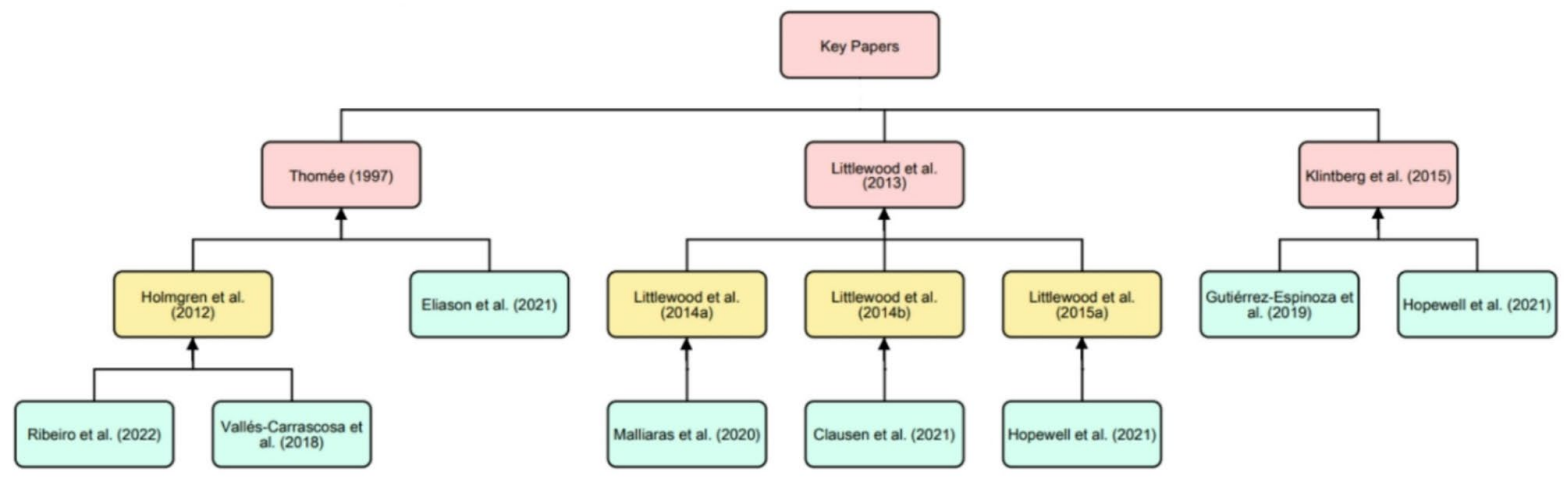
Citation tracking overview for the most cited papers The green boxes represent papers included in this review [49, 53, 56, 58, 66, 68, 72], and their relation to the key papers, through either direct or indirect (yellow boxes) citation

Among the referenced articles underpinning the chosen pain-related prescription parameters, various sources of evidence were identified, ranging from previous RCTs to single-subject research. A key feature for most cited sources of evidence was further referencing to other papers without clear justifications for the relevance of applied parameters. Backward tracking of provided citations revealed three key papers underpinning most chosen pain-related prescription parameters. By either direct or indirect citation, Thomée et al. [73], Littlewood et al. [74] and Klintberg et al. [18], were respectively identified as the most cited papers. The backward citation tracking process and related citations is described in Fig. 2.

**Fig. 3:**
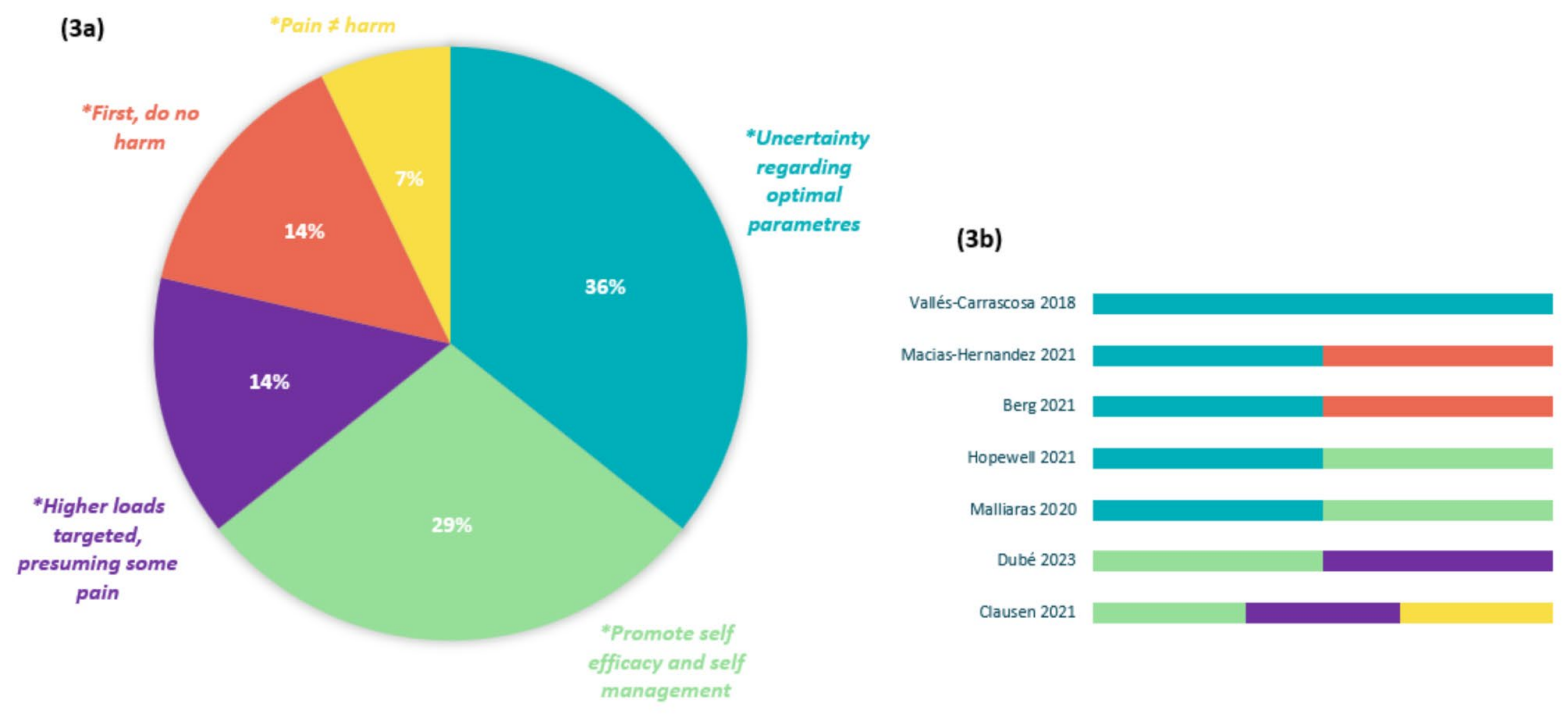
Justification themes (**3a**) and distribution of these themes (**3b**) across respective studies

Among the 28 RCTs, justifications for the chosen pain-related prescription parameters were provided in seven (25%) studies [47, 49, 52, 58, 65, 66, 72], and five distinct categories were developed consequent to inductive qualitative analysis. All seven studies providing any justification for their choice of prescription parameters were either positive or ambiguous in their stance on pain allowance. None of the studies explicitly discouraging painful exercise provided any justification or rationale for their chosen instructions. The identified justification themes and allocation of associated articles are illustrated in Fig. 3.

All studies providing justifications were assigned to two or more categories, with the exception of Vallés-Carrascosa et al. [72]. Among the seven studies that provided a rationale, five [47, 58, 65, 66, 72] justified their selection of pain-related prescription parameters by emphasizing the existing scientific uncertainty about the optimal parameters for RCRSP. Accordingly, two of those studies [47, 65] adopted the principle of “first, do no harm” in the context of scientific uncertainty, although both studies allowed some degree of pain in their exercise protocols. Conversely – also within the context of scientific uncertainty – two studies [58, 66] allowed some degree of pain with certain ground rules to “promote self-efficacy and self-management”. The study of Vallés-Carrascosa et al. [72] justified their choice of applying different pain-related prescription parameters to different interventions groups on the basis of “scientific uncertainty regarding optimal parameters”. The two remaining studies provided a mix of several justifications, themed as “promoting self-efficacy and self-management” [49, 52], “Higher loads targeted, presuming some pain” [49, 52] and “Pain ≠ harm” [49].

## Discussion

The aim of this scoping review was to investigate the reporting, implementation, and description of pain-related prescription parameters—including pain allowance and pain monitoring models—in clinical trials evaluating resistance exercise interventions for RCRSP. Additionally, it sought to explore the rationale and supporting evidence underlying the chosen parameters. This scoping review reveals substantial reporting deficiencies for pain-related prescription parameters. Analysis of the extracted data revealed a great degree of heterogeneity regarding applied parameters, pain allowance styles, and for reporting of citations and/or justifications for the chosen pain-related prescription parameters.

### Reporting of pain-related prescription parameters

The results from our review show insufficient reporting of pain-related prescription parameters in 67% of all studies remaining after the screening process. Inadequate details regarding exercise interventions is a well-known phenomenon that substantially impacts the ability of systematic reviews to conclude with specific details regarding effective exercise interventions [4]. Reporting templates like TIDieR and CERT were developed to address intervention reporting inadequacy in clinical trials, but recent studies evaluating adherence to these reporting guidelines in RCRSP-research suggest a persistent reporting inadequacy [26]. Pain-related prescription parameters are not explicit categories in TIDieR or CERT, which may contribute to the lack of detailed reporting on these parameters, as reflected in our results. Continuous refinement and identification of new reporting categories for CERT-guidelines has previously been suggested [28, 75]. The elaboration and explanation document for CERT indirectly implies that pain-related prescription parameters are incorporated in some of the 16 CERT-items [25], but our findings suggests that one or more explicit items for these parameters might be warranted.

Moreover only 46% of the included studies in this review reported some information about the use or prescription of analgesics. Analgesics use can be a confounding factor, potentially influencing replication of interventions and their associated outcomes [76]. Exercise progression informed by reported pain levels during exercise was the most employed pain-related progression criteria in this review. Analgesics use has the potential to influence an individuals’ pain perception, possibly allowing higher loads and exercise intensity. Conversely, long-term analgesic use has also been associated with increased pain sensitivity [77], possibly influencing the applicability of certain pain-related prescription parameters. Increased focus on clear reporting of analgesics-use is therefore warranted in future clinical trials, facilitating transferability and identification of mediating effects.

### Pain-allowance; a necessity for adequate loading?

The included studies used a wide range of pain-related prescription styles for their exercise interventions. Applied instructions ranged from discouraging painful exercise, to allowing or encouraging pain during exercise on the condition of symptom cessation within 24 h. This heterogeneity reflects the current knowledge in the field, as there seem to be no clear consensus or well-established evidence for the superiority of painful exercises compared to pain free exercises in the management of RCRSP [16, 18, 78].

Furthermore, 22 (79%) of the included studies in this review used pain intensity or pain response as a parameter to guide load and exercise progression; with the most common approach being progression of load once the previous step could be performed pain-free (32%), or within individual pain tolerance (18%). The development of exercise protocols allowing pain in RCRSP have been influenced by principles of dose-response in successful exercise protocols for lower limb tendinopathies [27, 79], where pain is partially allowed or encouraged in order to target higher levels of resistance or load [29]. Progressive resistance exercise has the potential to stimulate tensile strength, collagen production, reduce pain, and improve function in various tendon pathologies [80], but the clinical relevance of this dose-response relationship has not been established in RCRSP when comparing higher and lower exercise loads [49, 79]. In contrast to more tendon-specific diagnostic labels, like patellar -and achilles tendinopathy, RCRSP covers various diagnostic labels, underpinned by the limited tools for accurately isolating the painful structure(s) in the shoulder complex [6]. Consequently, identification of specific loading regimes for RCRSP, with the intent of targeting specific anatomical structures might be challenging in practice, compared to other tendinopathies. Exercise regimens targeting higher loads, with the intent of enhancing tendon-properties, do not necessarily align with the expectations of the individual – especially when there is pain involved. Not addressing this discrepancy when prescribing painful exercise might lead to maladaptive pain-behaviour, negatively impacting self-efficacy and exercise adherence [21].

As seen in the included studies of this review, pain-related prescription parameters are frequently used to guide progression and self-management of symptoms, but their value without concomitant patient education is questionable [81]. Recent evidence suggests that in exercise therapy, 46% of the overall pain relief can be attributed to contextual effects, implying multiple pathways to enhanced therapeutic outcomes [82].This might explain the absence of a clear causal relationship between higher training volumes, pathophysiological changes, and favorable outcomes in people with RCRSP. An exploration of contextual factors, such as pain-related prescription parameters, and their influence on treatment outcomes in RCRSP might therefore be warranted.

### Unidimensional pain scales

The results from this review show that NRS and VAS were the most applied scales for pain-monitoring during exercise interventions for RCRSP. These scales are well established instruments for pain measurement in both research and clinical practice guidelines for a wide range of different musculoskeletal conditions, and are described as valid, reliable and appropriate for their purpose [83]. Their popular applicability can partly be explained by minimal translation difficulties facilitating cross-cultural adaptations and the ability to quantitatively measure treatment effects and pain-related outcomes [84]. However, the frequent use of scales like VAS and NRS has also been criticized, as these scales reduce a highly complex and subjective phenomenon to a quantifiable measure, failing to illustrate the multifactorial processes influencing an individual’s pain experience [85].

### Pain limits; does one size fit all?

All of the included studies using pain scales applied static upper pain limits, with the sole exception of Ribeiro et al. [68] who applied a dynamic approach for their upper pain limit during exercise, permitting a three point increase from habitual pain levels. In acute stages of RCRSP, activity restriction (like the use of a static upper limit for pain intensity) can be a reasonable approach, in order to stabilize potential inflammatory processes, appropriately manage irritable clinical presentations and avoid excessive exacerbation of symptoms [86]. However, all included studies prohibiting painful exercise or applying static upper limits for pain allowance included participants with either subacute or chronic pain.

Persistent RCRSP has been associated with fear-avoidance behaviour, catastrophizing beliefs and a range of other psychosocial factors, contributing to maladaptive pain behaviour [87]. Furthermore, the involvement of central pain sensitization is believed to be a potential driver for persisting RCRSP, which might imply poor correlation between pain intensity and actual tissue damage [29, 88]. While intended to provide safety, using a static upper limit such as 3/10 NRS or advising against pain during exercise may unintentionally reinforce maladaptive pain behaviour, and ultimately become a barrier to exercise adherence and self-efficacy in certain clinical presentations. When managing persistent RCRSP, individually tailored pain monitoring strategies, balancing safety with confidence in movement and gradual exposure to tolerable discomfort may therefore enhance adherence and optimize results.

In this review, eight (29%) studies out of 28 RCTs applied an “individualized” pain limit, allowing patients to determine what they perceived as an acceptable pain response to load. Transitioning from strict, generic biomechanical models for pain management, to pragmatic individualized approaches has been advocated in order to address the complexity of RCRSP [16, 87]. This view is reflected in qualitative syntheses of patients’ experience on exercising with RCRSP, where pain education and individualized load management are highlighted as key factors for a successful self-managed rehabilitation [19]. Consequently, individually tailored load management strategies can facilitate reconceptualization of the individual’s pain experience regardless of applied pain tolerance limits during exercise. Within this framework, establishing an upper or lower numerical limit for pain permission during exercise might not be necessary, as it has been suggested that individuals can successfully self-manage acceptable pain levels in exercise interventions for RCRSP [81]. However, most patient education seems to base its content on traditional pain models, reinforcing the idea of specific biomechanical mechanisms being the primary driver for pain [19]. Addressing the individual’s narrative regarding pain-drivers should therefore be considered when prescribing exercises where pain is allowed, to prevent inadvertently promoting pain catastrophizing [89].

### Pain-allowance; a pragmatic or evidence-based concept?

Among the included studies in this review, only 36% provided references or justifications for their applied pain-monitoring parameters, with five main themes for justifications and three key papers emerging (as described in Figs. 2 and 3). Interestingly, only studies allowing some degree of pain during exercise provided justifications for their chosen pain-related prescription parameters. The most common justification revolved around the scientific uncertainty regarding optimal pain-related prescription parameters, resulting in pragmatic approaches based on clinical reasoning and previously published experimental trials. *Promoting self-efficacy and self-management* through pain allowance was the other prominent justification, reflecting newly proposed paradigms in musculoskeletal pain management [29, 90]. Justifications for explicitly discouraging painful exercise could not be found based on our results, with only one study [56] proving a citation for this choice by referencing an expert consensus statement. The practice of being overly restrictive in regard to pain allowance, and its underlying rationale should therefore be elaborated in future trials.

As for the three key papers that were commonly referenced for pain-related prescription parameters; three distinct approaches were identified. Thomée [73], cited directly or indirectly by three of the included papers, designed the *pain monitoring system* while developing a comprehensive treatment approach for Patellofemoral pain syndrome in young women. The *pain monitoring system* is based on a VAS scale, with the values < 2, 2–5 and > 5 representing safe, acceptable and high-risk zones on the condition that pain subsides by the next morning. The limits and pain scale were chosen based on the authors’ own clinical experience, and have since been adapted as a pain monitoring model in a range of influential RCT studies on achilles, patellar and rotator cuff tendinopathy [91–93]. To our knowledge, no study has evaluated the limits proposed in the *pain monitoring system* compared to other values or other pain monitoring models, despite its frequent use in musculoskeletal research and clinical practice.

Another key paper, by Littlewood et al. [74], cited directly or indirectly by three of the included studies, describes the development of a self-managed loaded exercise program for rotator cuff tendinopathy. Self-monitoring of symptoms, and individual judgement of what constitutes an acceptable symptom response are regarded as foundations for successful self-management within their exercise program. They avoid the use of static upper limits for pain intensity, as it can potentially limit progression for individuals tolerating higher pain intensity whilst exercising. The entire program revolves around promoting self-efficacy through knowledge translation, individual involvement and feedback/persuasion. These principles are in line with a common justification for pain allowance reported in several of the included studies [49, 52, 58, 66].

Finally, the last key paper by Klintberg et al. [18], cited directly by two of the included studies, describes the development of an expert consensus-based treatment algorithm for RCRSP. For pain-related prescription parameters, no consensus was reached, but two different approaches emerged: (1) Selecting exercises that would not reproduce familiar pain and (2) Some localized pain ≤ VAS 4/10 should be allowed during exercise, on the condition of symptom cessation within a short timeframe. The different approaches described in this treatment algorithm are predominantly based on the clinical expertise and experience of leading experts within the field, and no additional supporting evidence for the chosen limits was provided.

In summary, the three key papers revealed different approaches to pain allowance during exercise, reflecting the lack of consensus and supporting evidence regarding pain-related prescription parameters reported in the included studies of this review. Cross-sectional studies surveying current physiotherapy practice have revealed a similar variance in applied pain-related prescription parameters [15, 31]. To enhance patient-centered care, a pragmatic and individualized approach to pain allowance might bridge the gap between approaches advocating strict pain prohibition and static pain intensity limits with little added context.

### Strengths and limitations

In this study, we adhered to best-practice guidelines, ensuring clearly reported and well-defined methodology throughout the process. The search process was overviewed by a librarian with relevant expertise, and the screening process was performed by two independent reviewers blinded to each other to avoid selection bias.

The search was restricted to studies only published in English, which could have increased the risk of language and publication bias. The included studies did however represent research from 17 different countries. An excessive limitation to eligibility criteria, such as the inclusion of only RCTs, is generally uncommon in scoping reviews but was deemed appropriate given the context of our research objectives. However, we acknowledge that relevant insights into prescription parameters and their underlying justifications may also be derived from other study designs, possibly enhancing the findings by providing a broader perspective and theoretical underpinnings. A limitation of this study is the restriction of study inclusion to the 2018–2023 timeline. While this timeframe was selected to ensure a focus on recent advancements in the field, it inevitably led to the exclusion of a considerable number of studies published prior to 2018 (*n* = 118, Fig. 1). This restriction may have influenced the comprehensiveness of the findings, and future research could benefit from adopting a broader temporal scope to capture a more extensive body of relevant literature.

To broaden the scope of the review, we also included studies that lacked an exercise-only arm. Among the included studies, resistance exercise was frequently administered alongside other modalities, which might have resulted in less extensive reporting of the exercise intervention in these clinical trials. Ultimately, this can explain why only one third of the studies remaining after the screening process reported any pain-related prescription parameters. The included studies were not critically appraised with regards to quality and risk of bias [32] as this is not customary in scoping review methodology, and the results should be interpreted with this in mind.

In this scoping review, the properties of different pain-related prescription parameters are discussed and challenged, but we emphasize that our methodology does not permit inferences regarding the effectiveness and superiority of specific pain-related prescription parameters.

## Conclusion

This scoping review reveals considerable reporting deficiencies for pain-related prescription parameters in RCTs involving resistance exercises for RCRSP. Various pain-related prescription parameters were identified, ranging from discouraging painful exercise to allowing or even encouraging it. Differing pain limits were applied, including numerical values and individual tolerance, imposed at different time frames relative to exercise sessions. Moreover, only a minority of studies provided references or justifications for their applied pain-related prescription parameters, indicating a lack of consensus or evidence-based guidance underpinning these parameters. Pain-related prescription parameters seem to be predominantly pragmatic in nature, founded on clinical expertise rather than research evidence. Future trials comparing individually tailored and standardized approaches to pain allowance in exercise prescription may offer clarity and valuable knowledge. Our findings suggest that reporting guidelines should incorporate clearer instructions regarding pain-related prescription parameters, facilitating interpretation and implementation into clinical practice.

## Electronic supplementary material

Below is the link to the electronic supplementary material.

Additional file 1: A document depicting the primary search strategy in all databases is provided to ensure research transparency and possibilities for replication of searches

Additional file 2: A list of the studies passing the initial screening process, but were excluded prior to data extraction due to the lack of reported pain-related prescription parameters

Additional file 3: An overview of the respective PEDro scores of included studies, according to the PEDro scale

## Data Availability

The datasets used and/or analyzed during the current study that are not included in the additional files will be made available from the corresponding author upon reasonable request.

## Abbreviations

ACSM: American College of Sports Medicine
CERT: Consensus on Exercise Reporting Template
CINAHL: Cumulative Index to Nursing and Allied Health Literature
JBI: Joanna Briggs Institute
NRS: Numerical Rating Scale
OSF: Open Science Framework (OSF)
PEDro: Physiotherapy Evidence Database
PCC: Population, Concept, and Context
PRISMA-ScR: Preferred Reporting Items for Systematic Reviews and Meta-Analyses extension for Scoping Reviews
RCT: Randomized Controlled Trial
RCRSP: Rotator Cuff Related Shoulder Pain
ROM: Range of Motion
TIDieR: Template for Intervention Description and Replication
VAS: Visual Analogue Scale
