# Supplementary material for: Painful considerations in exercise-management for rotator cuff related shoulder pain: a scoping review on pain-related prescription parameters
Source: BMC Musculoskelet Disord. 2025 Feb 22;26:180. doi: 10.1186/s12891-025-08411-7 (PMC11846222; doi:10.1186/s12891-025-08411-7)

##### **Additional file 1: Search strategy across different databases**

The primary search strategy in all databases is provided below for transparency and to provide possibilities for replication of searches. The limitation to publication dates within the last 10 years were used in all databases, including records published from 1/1/13- 5/12/23 (date of final search). Despite only including studies from the last 5 years in the screening process, a filter starting from 2013 was applied in the primary search, to include study registries submitted before 2018. This decision was taken for the scenario where studies were indexed with multiple or incorrect date entries (e.g. studies that were indexed with their protocol registration date entry only, especially prevalent in the Central (Cochrane) database).


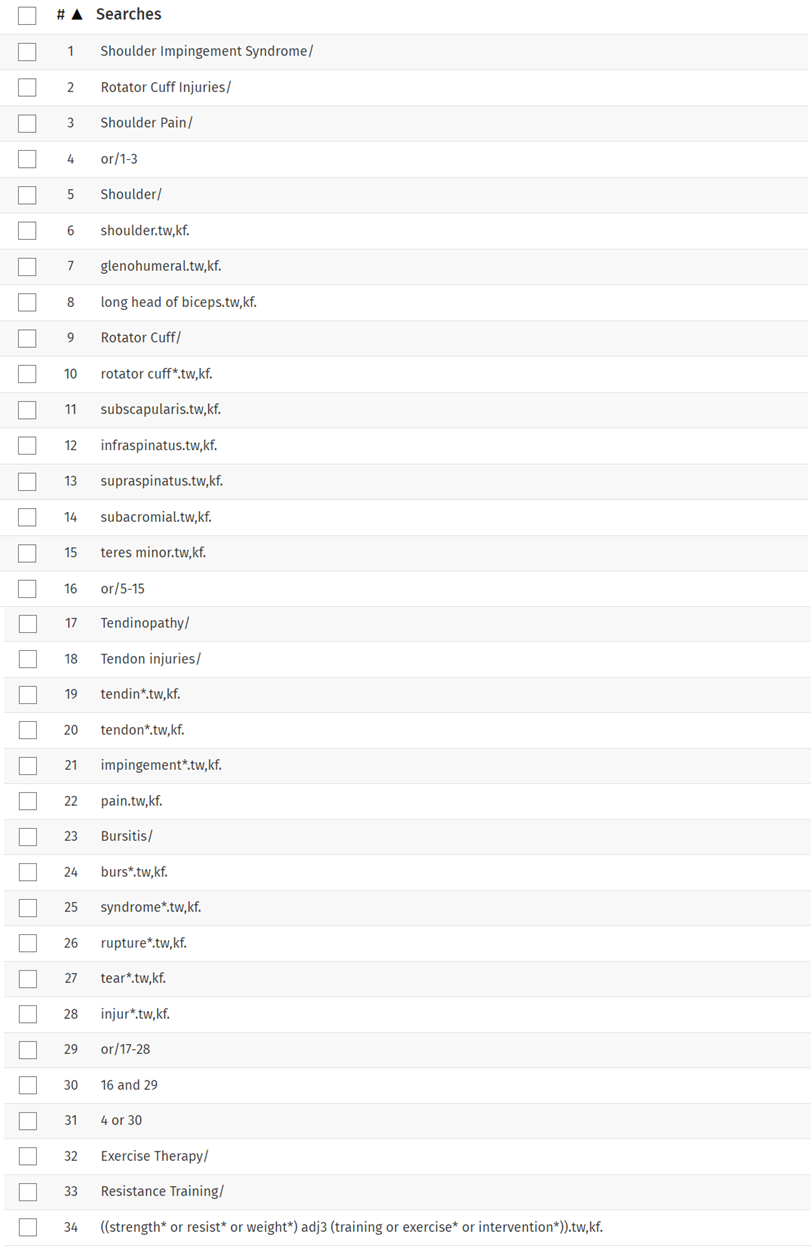


## Medline (Ovid):

######
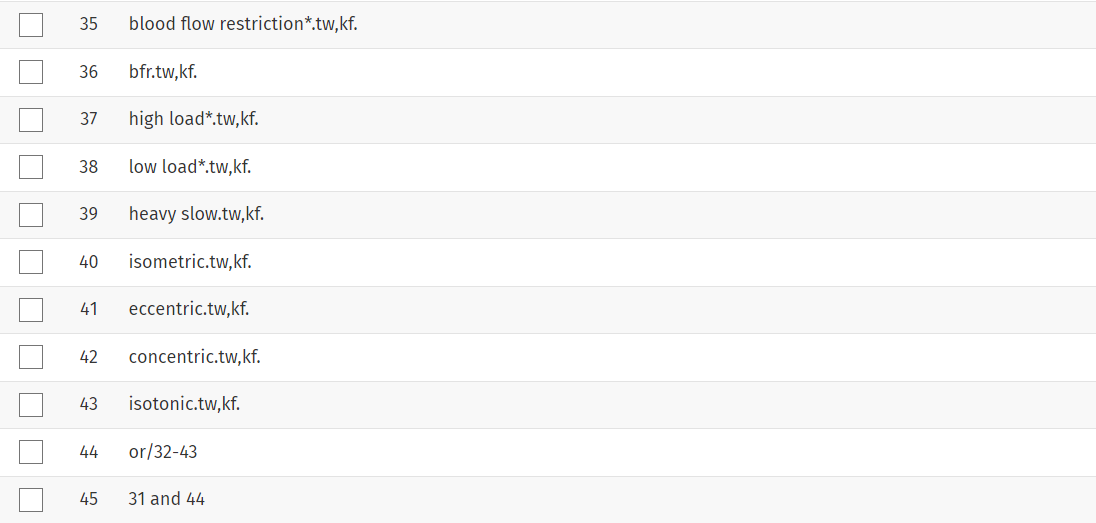


## Medline (Embase):


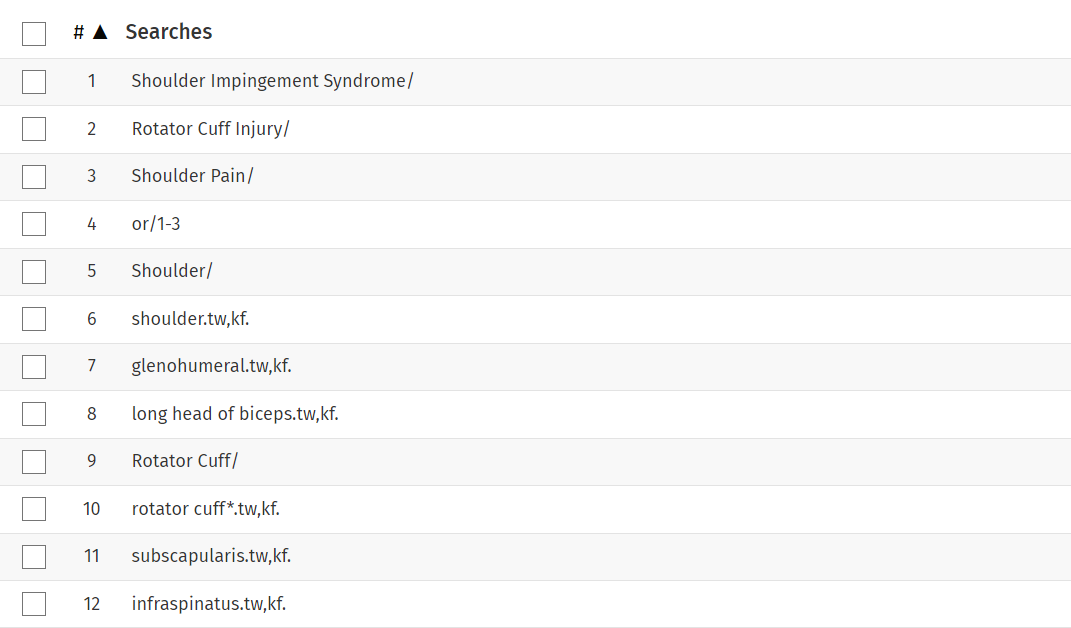


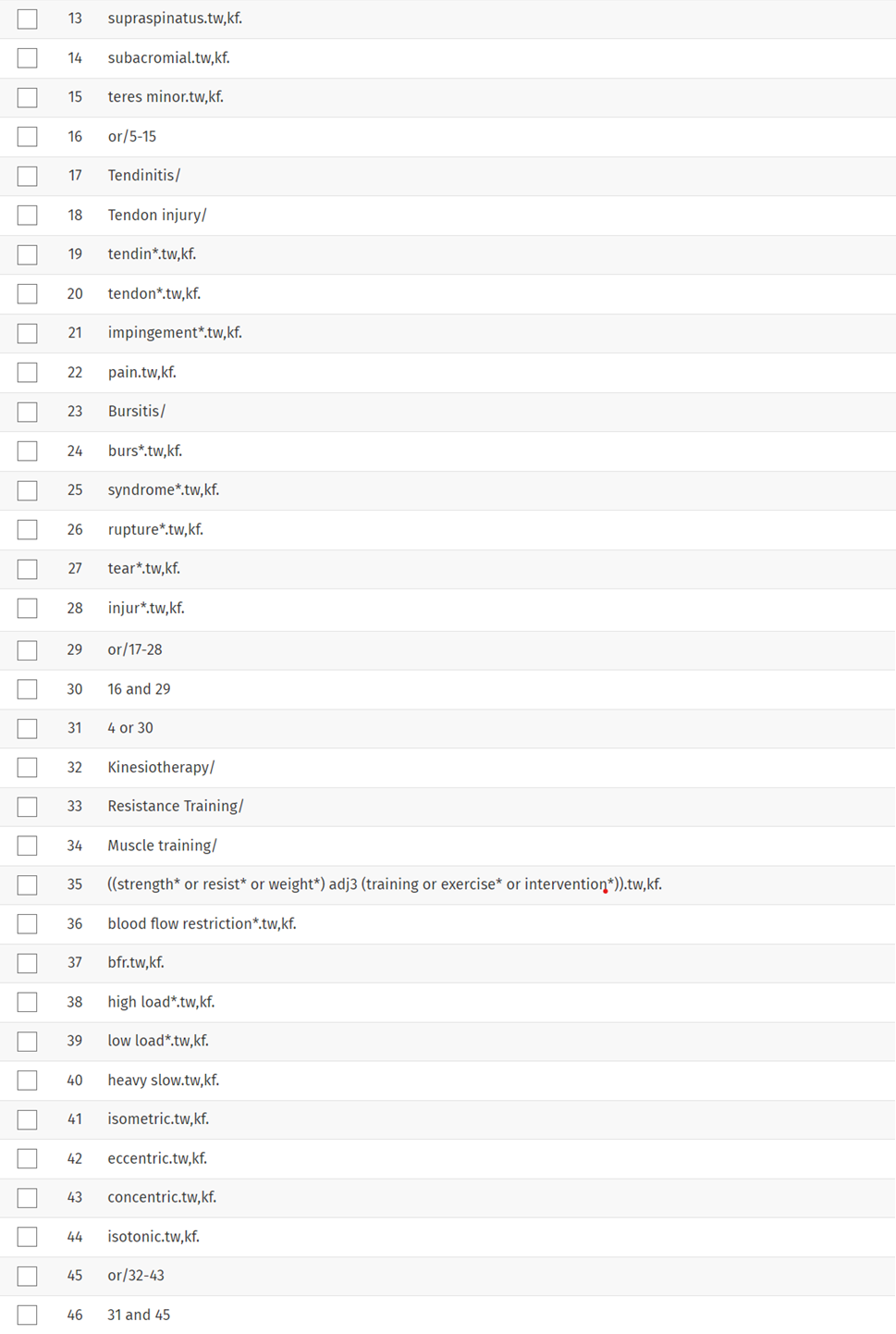


## Cinahl (EBSCO):


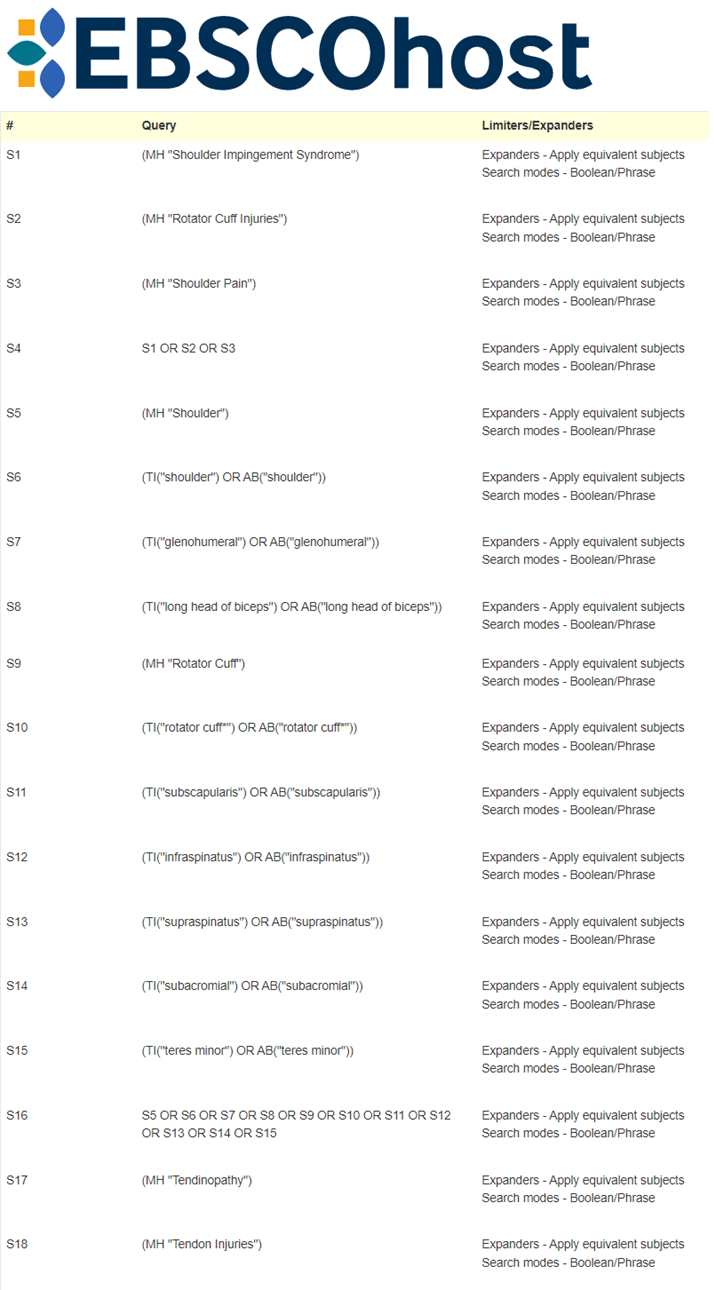


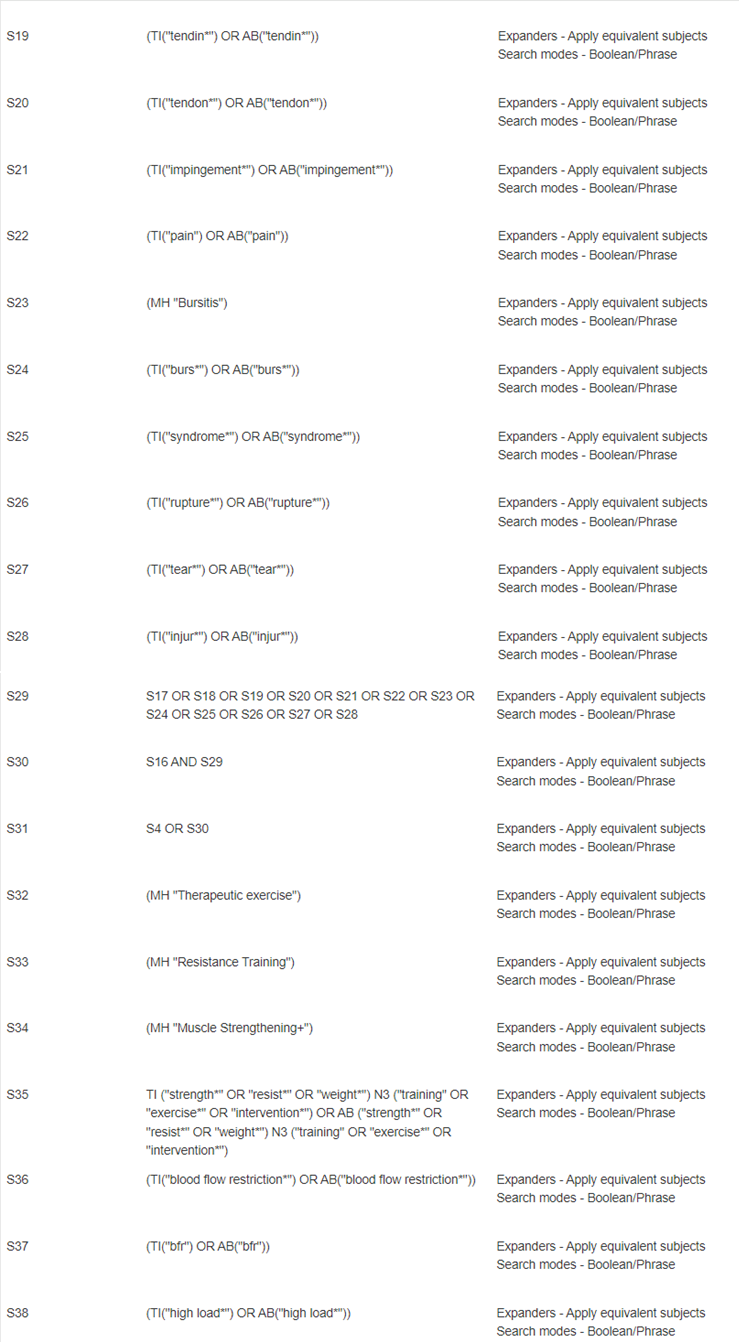


######
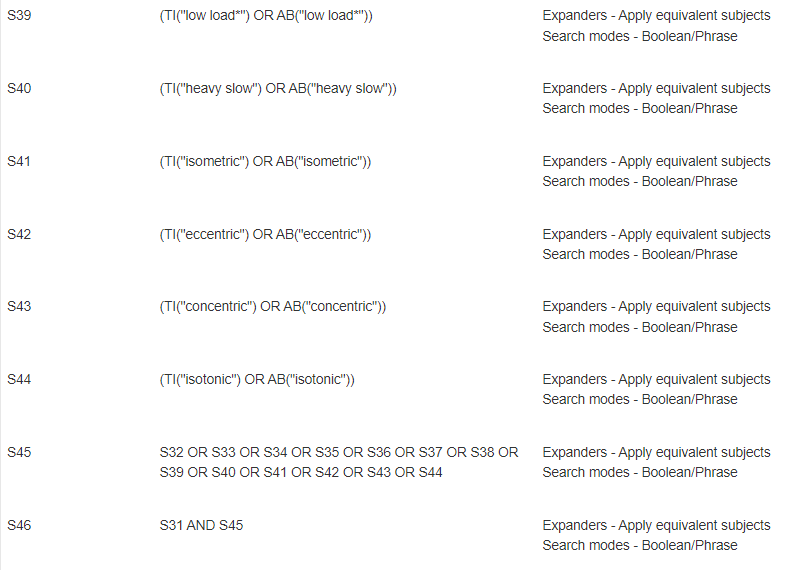


## Central (cochrane)

##
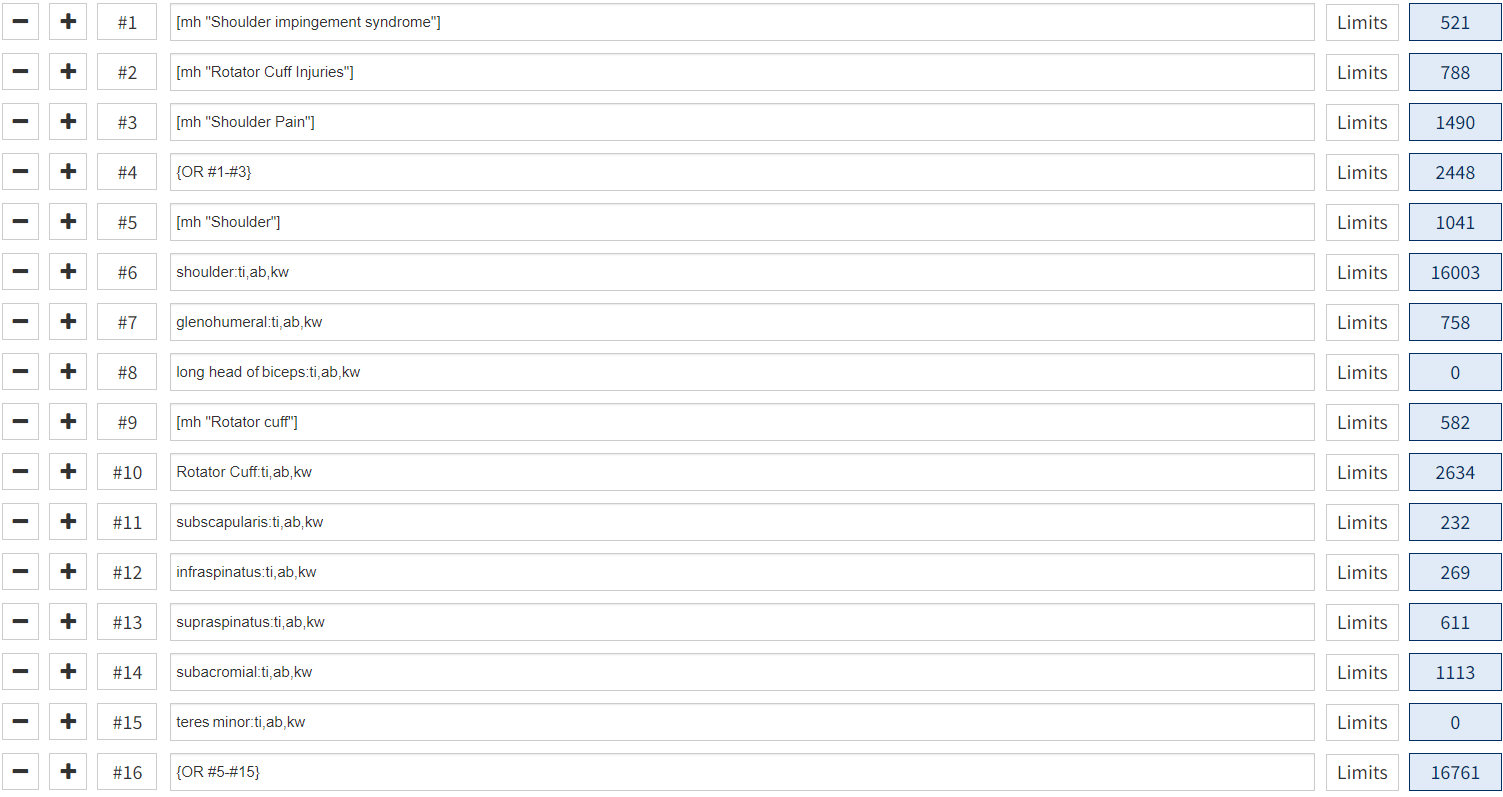


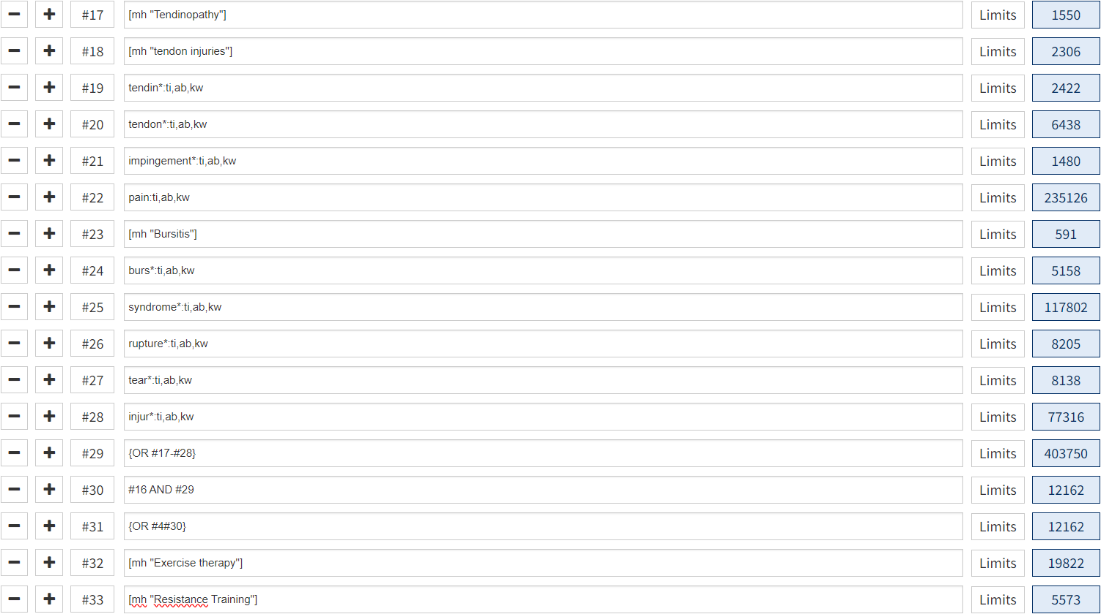


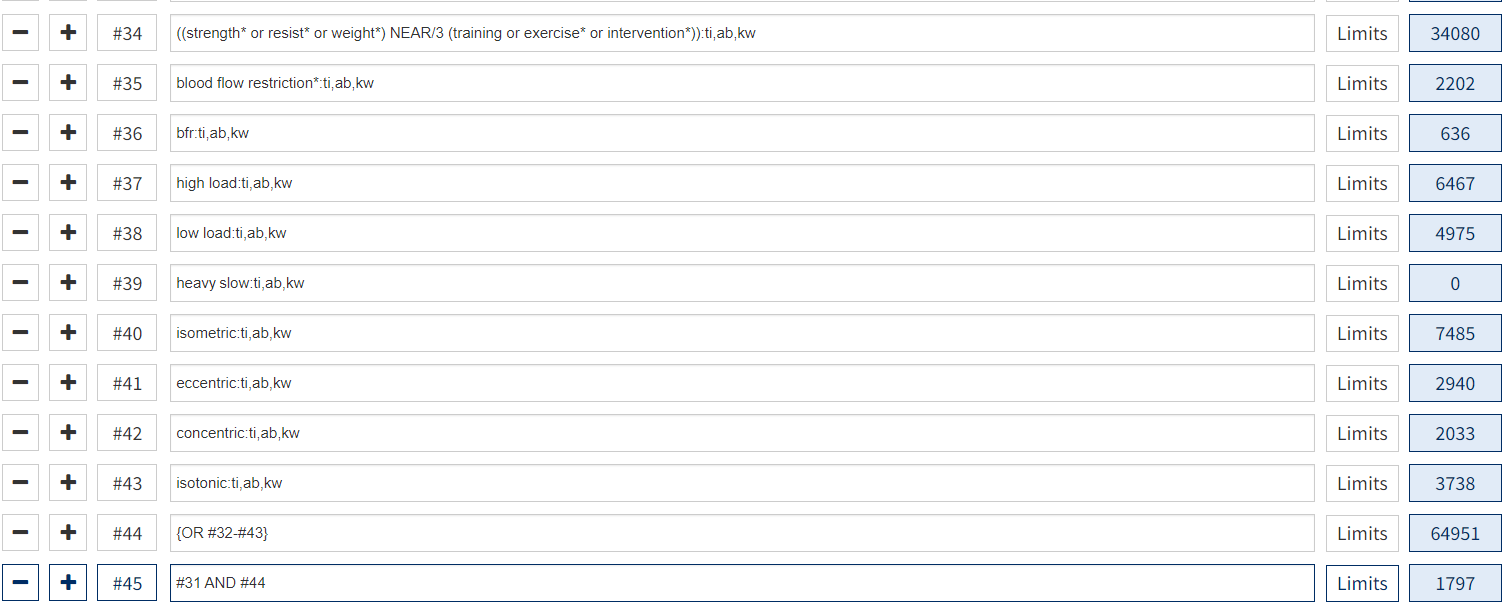


## PEDro

PEDro has a simpler search engine than the other included databases. We used the following word combination in PEDro: “Shoulder and pain and exercise”.


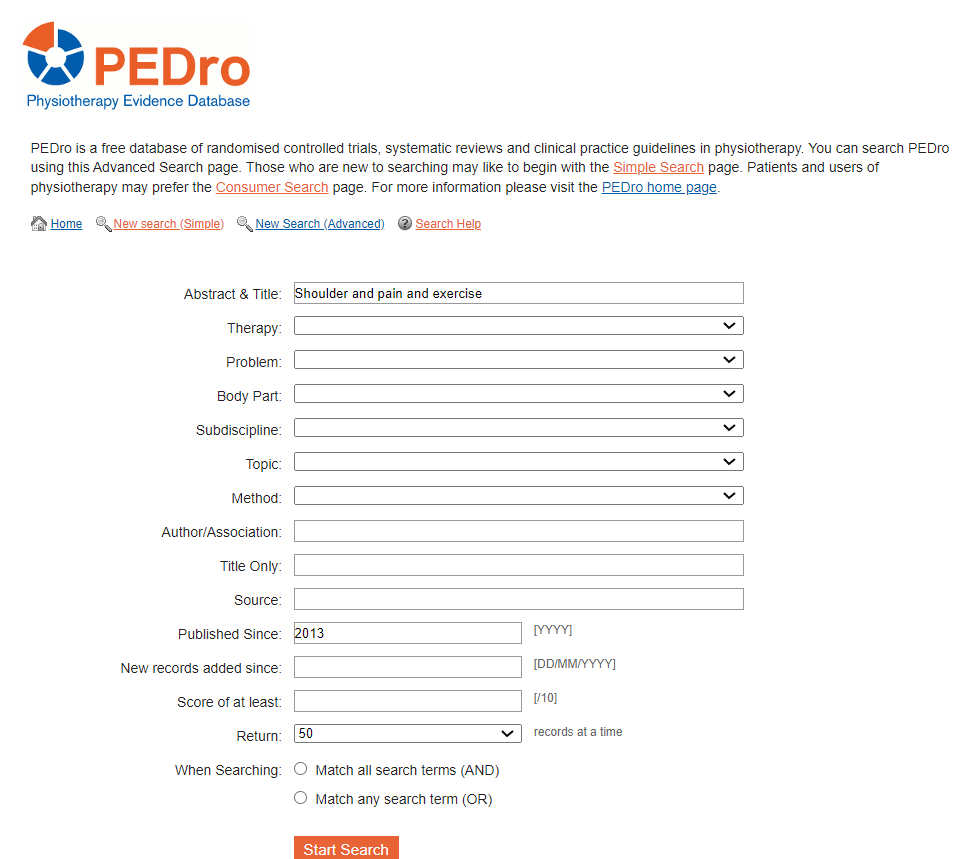

Supplement: Supplementary file 1 — Additional file 1: A document depicting the primary search strategy in all databases is provided to ensure research transparency and possibilities for replication of searches [file 12891_2025_8411_MOESM1_ESM.docx]
