# Supplementary material for: Painful considerations in exercise-management for rotator cuff related shoulder pain: a scoping review on pain-related prescription parameters
Source: BMC Musculoskelet Disord. 2025 Feb 22;26:180. doi: 10.1186/s12891-025-08411-7 (PMC11846222; doi:10.1186/s12891-025-08411-7)
Supplement: Supplementary file 3 — Additional file 3: An overview of the respective PEDro scores of included studies, according to the PEDro scale [file 12891_2025_8411_MOESM3_ESM.docx]

**Additional file 3**: PEDro score-assessment overview

| **Record** | **Score** | **Methodological**  **Quality** | **PEDro item number** | | | | | | | | | | |
| --- | --- | --- | --- | --- | --- | --- | --- | --- | --- | --- | --- | --- | --- |
|  |  |  | 1 2 3 4 5 6 7 8 9 10 11 | | | | | | | | | | |
| Ager et al. 2019 | 8 | Good  (Pilot RCT) | 1* | 1 | 1 | 1 | - | - | 1 | 1 | 1 | 1 | 1 |
| Belley et al. 2018 | N/R | - | - | - | - | - | - | - | - | - | - | - | - |
| Berg et al. 2021 | 6 | Good | - | 1 | 1 | 1 | - | - | - | - | 1 | 1 | 1 |
| Bourdreau et al. 2019 | 8 | Good | 1* | 1 | 1 | 1 | - | - | 1 | 1 | 1 | 1 | 1 |
| Clausen et al. 2021 | 8 | Good | 1* | 1 | 1 | 1 | 1 | - | 1 | - | 1 | 1 | 1 |
| DeOliveira AKA et al. 2022 | 8 | Good | - | 1 | 1 | 1 | - | - | 1 | 1 | 1 | 1 | 1 |
| DeOliveira FCL et al.  2021 | 8 | Good | 1* | 1 | 1 | 1 | - | - | 1 | 1 | 1 | 1 | 1 |
| Dubé et al.  2023 | 8 | Good | 1* | 1 | 1 | 1 | - | - | 1 | 1 | 1 | 1 | 1 |
| Eliason et al. 2021 | 8 | Good | 1* | 1 | 1 | 1 | - | - | 1 | 1 | 1 | 1 | 1 |
| Eraslan et al. 2023 | 7 | Good | 1* | 1 | 1 | 1 | - | - | 1 | 1 | - | 1 | 1 |
| Gomes et al. 2018 | 8 | Good | 1* | 1 | 1 | 1 | - | - | 1 | 1 | 1 | 1 | 1 |
| Gutiérrez-Espinoza et al. 2019 | 8 | Good | 1* | 1 | 1 | 1 | - | - | 1 | 1 | 1 | 1 | 1 |
| Gutiérrez-Espinoza et al. 2023 | 8 | Good | 1* | 1 | 1 | 1 | - | - | 1 | 1 | 1 | 1 | 1 |
| Hopwell et al. 2021 | 6 | Good | 1* | 1 | - | 1 | - | - | - | 1 | 1 | 1 | 1 |
| Hui et al. 2023 | 8 | Good (Pilot RCT) | 1* | 1 | 1 | 1 | - | - | 1 | 1 | 1 | 1 | 1 |
| Juul-Kristensen et al. 2019 | 8 | Good | 1* | 1 | 1 | 1 | - | - | 1 | 1 | 1 | 1 | 1 |
| Kang et al. 2019 | 7 | Good | 1* | 1 | 1 | 1 | - | - | 1 | 1 | - | 1 | 1 |
| Karaaslan et al. 2023 | 7 | Good | 1* | 1 | 1 | 1 | - | - | 1 | 1 | - | 1 | 1 |
| Kim et al. 2020 | 6 | Good | 1* | 1 | - | 1 | - | - | - | 1 | 1 | 1 | 1 |
| Letafatkar et al. 2021 | 8 | Good | 1* | 1 | 1 | 1 | - | - | 1 | 1 | 1 | 1 | 1 |
| Macías-Hernandez et al. 2021 | 7 | Good (Pilot RCT) | 1* | 1 | 1 | 1 | - | - | 1 | 1 | - | 1 | 1 |
| Malliaras et al. 2020 | 4 | Fair  (pilot/feasibility RCT) | 1* | 1 | 1 | - | - | - | - | 1 | - | - | 1 |
| Martins Da Silva et al. 2020 | 7 | Good | - | 1 | 1 | 1 | - | - | 1 | 1 | - | 1 | 1 |
| Riberio et al. 2022 | 7 | Good  (feasibility RCT) | 1* | 1 | 1 | 1 | - | - | 1 | 1 | 1 | - | 1 |
| Rodriguez-Huguet et al. 2020 | N/R | - | - | - | - | - | - | - | - | - | - | - | - |
| Santello et al. 2020 | 8 | Good | 1* | 1 | 1 | 1 | - | - | 1 | 1 | 1 | 1 | 1 |
| Subbiah et al. 2023 | CBR | - | - | - | - | - | - | - | - | - | - | - | - |
| Vallés-Carrascosa et al. 2018 | 7 | Good | 1* | 1 | 1 | 1 | - | - | - | 1 | 1 | 1 | 1 |

**Abbreviations**: *: Eligibility criteria item that does not contribute to total score, N/R: Not rated, CBR: Currently being rated in PEDro.
